# Letrozole Versus Clomiphene Citrate and Natural Cycle: Endometrial Receptivity During Implantation Window in Women With Polycystic Ovary Syndrome

**DOI:** 10.3389/fendo.2020.532692

**Published:** 2021-01-18

**Authors:** Li Wang, Shulan Lv, Fen Li, E. Bai, Xiaofeng Yang

**Affiliations:** Department of Obstetrics and Gynecology, The First Affiliated Hospital of Xi’an Jiaotong University, Xi’an, China

**Keywords:** polycystic ovary syndrome, letrozole, clomiphene citrate, endometrial receptivity, embryo implantation

## Abstract

**Objective:**

Numerous studies have reported on ovulation and pregnancy rates in patients with polycystic ovary syndrome (PCOS). However, relevant data on endometrial receptivity are limited. This study was conducted to compare endometrial receptivity during implantation windows among letrozole (LE), clomiphene citrate (CC), and natural cycle, and to assess the predictive value for pregnancy of observed indicators.

**Methods:**

This randomized controlled trial study enrolled 270 patients with PCOS. Patients were given LE (n=90) at a dose of 2.5mg/day or CC (n=90) at a dose of 50 mg/day on cycle days 5–9 for ovulation induction. Patients in the natural cycle group (n=90) did not receive any drug for ovulation induction. Endometrial ultrasonic parameters, integrin αvβ3, and vascular endothelial growth factor (VEGF) concentrations in uterine secretion were detected during the implantation window. The endometrial receptivity, ovulation rate, pregnancy rates, and predictive value of observed indicators for pregnancy were analyzed.

**Results:**

The successful ovulation rate did not differ between the LE group and CC group (*P*>0.05). Endometrial ultrasonic parameters [endometrial thickness (ET), endometrial volume (EV), vascularization index (VI), flow index (FI), vascularization flow index (VFI)], integrin αvβ3, and VEGF concentrations in uterine fluid were significantly higher in the LE group compared with the CC group and natural cycle group (*P*<0.05). The clinical pregnancy and ongoing pregnancy rates of the LE group were significantly higher than in the CC group (*P*<0.05). Endometrial ultrasonic parameters (VI, FI, and VFI), integrin αvβ3, and VEGF concentrations in uterine fluid of all pregnancy groups were significantly higher compared with the no pregnancy group (*P*<0.05), and the above parameters in ongoing pregnancy were significantly higher than in biochemical pregnancy (*P*<0.05). The endometrial FI during the implantation window had the highest predictive value for pregnancy (AUC=0.889). The integrin αvβ3 in uterine fluid had better predictive value (AUC=0.876) than VEGF.

**Conclusions:**

Endometrial receptivity during the implantation window of LE is superior to CC in PCOS women, which may be related to higher clinical pregnancy and ongoing pregnancy rates. Endometrial FI examined by 3-D power Doppler, and integrin αvβ3 in uterine secretion during the implantation window, could be preferable non-invasive predictor markers for pregnancy.

**Clinical Trial Registration:**

www.chictr.org.cn, ChiCTR1900023423.

## Introduction

Polycystic ovary syndrome (PCOS) is an endocrine and metabolic disorder in women of reproductive age, and its prevalence rate is from 9% (NIH criteria) to 18% (Rotterdam criteria) (1). The clinical manifestations of PCOS are menstrual irregularities, anovulatory infertility, hyperandrogenism, and disorders of glucose and lipid metabolism (2, 3). About 25%–30% of PCOS women of reproductive age need to seek help because of ovulatory dysfunction infertility (4). Clomiphene citrate (CC) promotes follicular development through blocking the negative feedback of estrogen to the hypothalamus and making the pituitary secrete gonadotropin. Therefore, CC has been used as the traditional first-line medication for inducing ovulation in PCOS women, but the antiestrogenic effect of CC on cervical mucus and endometrial receptivity results in low pregnancy rates (5). Letrozole (LE) was initially applied to treat breast cancer through preventing the conversion of androgens to estrogen and reducing the level of estrogen in the body. As a result, gonadotropin secretion increases due to blocking estrogen-negative feedback of LE, which stimulates the development of ovarian follicles (6).

Endometrial receptivity is critical for embryo implantation, and its impairment has been proven to be an important factor for infertility (7). In recent years, ultrasonic parameters, molecular markers in endometrial tissue and uterine secretions, endometrial microstructure, and hysteroscopy have been applied to evaluate endometrial receptivity (8). The preferred method for assessing endometrial receptivity is transvaginal ultrasound, and multiple ultrasonic indicators have been used to assess endometrial receptivity (9).

Numerous studies have reported on ovulation and pregnancy rates between LE and CC in PCOS women. However, relevant data on endometrial receptivity during an implantation window are limited, especially the non-invasive methods of assessment. Because LE and CC have different mechanisms for ovulation induction, we presumed that their endometrial receptivity during implantation windows is different, which affect subsequent pregnancy. The aim of this study was designed to compare the endometrial receptivity among an LE group, CC group, and natural cycle group, and to assess the predictive value for pregnancy of observed indicators.

## Materials and Methods

### Study Design

We conducted a randomized controlled trial (RCT) study on PCOS patients with infertility in the outpatient clinic of obstetrics and gynecology and women’s health care. All participants gave written informed consent on the basis of procedures granted by the Ethics Committee of The First Affiliated Hospital of Medical College of Xi’an Jiaotong University (XJTU1AF2019LSK-120). This study has been registered on China’s clinical trials registration: www.chictr.org.cn (ChiCTR1900023423).

### Participants

Two hundred and seventy-three PCOS patients were enrolled in the First Affiliated Hospital of Xi’an Jiaotong University from May 2018 to August 2019; among them three patients who declined to participate were excluded. All patients were between the ages of 22 and 38 years. The diagnostic criteria of PCOS was based on a modified Rotterdam criteria: menstrual abnormalities (oligomenorrhea, amenorrhea, or irregular uterine bleeding), combined with either hyperandrogenism or polycystic ovarian morphology (10). Hyperandrogenism was diagnosed according to either clinical manifestations or laboratory evidence. Clinical manifestations of hyperandrogenism included obesity or hirsutism. Obesity was defined as body mass index (BMI) ≥30.0 kg/m^2^ (11). Hirsutism was defined as a Ferriman–Gallwey score of more than 6 in physical examination (12). Laboratory evidence of hyperandrogenemia was defined as total testosterone level exceeding the upper limit of normal levels on the basis of local laboratory criteria. Polycystic ovarian morphology was defined as the following criteria: the presence of at least 12 antral follicles measuring 2-9 mm in diameter in unilateral ovary or bilateral ovaries, and (or) an increased ovarian volume (≥10 ml). Ovarian volume = 0.5 × length diameter × transverse diameter × anteroposterior diameter (10). Patients were ruled out if they had congenital adrenal hyperplasia (CAH), thyroid dysfunction, abnormal coagulation indicators, autoimmune disease or abnormal immune-related indicators, fallopian tube blocking, or a husband with abnormal semen. The baseline characteristics of all participants were recorded in detail. Serum sex hormone and anti-Mullerian hormone (AMH) concentrations on 2–4 days of menses were tested.

### Intervention

Patients were pretreated with lifestyle interventions, improved hyperandrogenism, hyperinsulinemia, and insulin resistance. Then 270 patients were randomly divided into three groups according to computer-generated random numbers, with 90 patients in each group. Patients in the LE group received LE for ovulation induction (2.5mg/day on cycle days 5–9 of menses for 1 cycle), and patients in the CC group received CC for ovulation induction (50 mg/day on cycle days 5–9 of menses for 1 cycle). Patients in the natural cycle group did not receive any drug for ovulation induction.

### Outcome Measures

The follicle and endometrial thickness (ET) were detected using transvaginal ultrasound. Human chorionic gonadotropin (hCG) at a dose of 5000–10000 IU was used to trigger ovulation when a dominant follicle appeared (the average diameter ≥18 mm). The indicators of endometrial receptivity of patients with successful ovulation in the three groups were tested 7 to 9 days after ovulation (defined as embryo implantation window period). Color Doppler was used to test resistance index (RI) and pulsatility index (PI) of the uterine artery. Additionally, 3-D power Doppler ultrasonography was used to calculate endometrial volume (EV), flow index (FI), vascularization index (VI), and vascularization flow index (VFI) (13). Meanwhile, uterine secretions were obtained using an embryo transfer catheter, which was linked to a syringe equipped with sodium chloride. The fluid was gently injected into the uterine cavity and then gently aspirated into the syringe (14). Integrin αvβ3 and vascular endothelial growth factor (VEGF) concentrations in the uterine fluid were tested using an enzyme-linked immunosorbent assay.

The diagnostic criteria for pregnancy were as follows: (a) serum hCG level exceed 10 mIU/mL tested at 2 weeks after ovulation was considered to be biochemical pregnancy; (b) appearance of pregnant bursa or embryo in the uterine cavity was considered to be clinical pregnancy; and (c) appearance of a fetus with a heartbeat at 12 weeks of pregnancy was considered to be ongoing pregnancy (4).

The primary outcomes were endometrial receptivity and predictive value for pregnancy of observed parameters. The secondary outcomes were follicular development, ovulation, and pregnancy rates.

### Statistical Analysis

Statistical analyses were performed using SPSS version 20.0. Normally distributed continuous data were given as mean ± standard deviation, which were analyzed by the analysis of variance or Student’s *t*-test. The enumeration variables were presented as number and percentage (%), which were analyzed using chi-square test or Fisher’s exact test. Endometrial receptivity of observed parameters was evaluated using receiver operating characteristic (ROC) curves. *P*< 0.05 was considered statistically significant.

## Results

**Figure 1:**
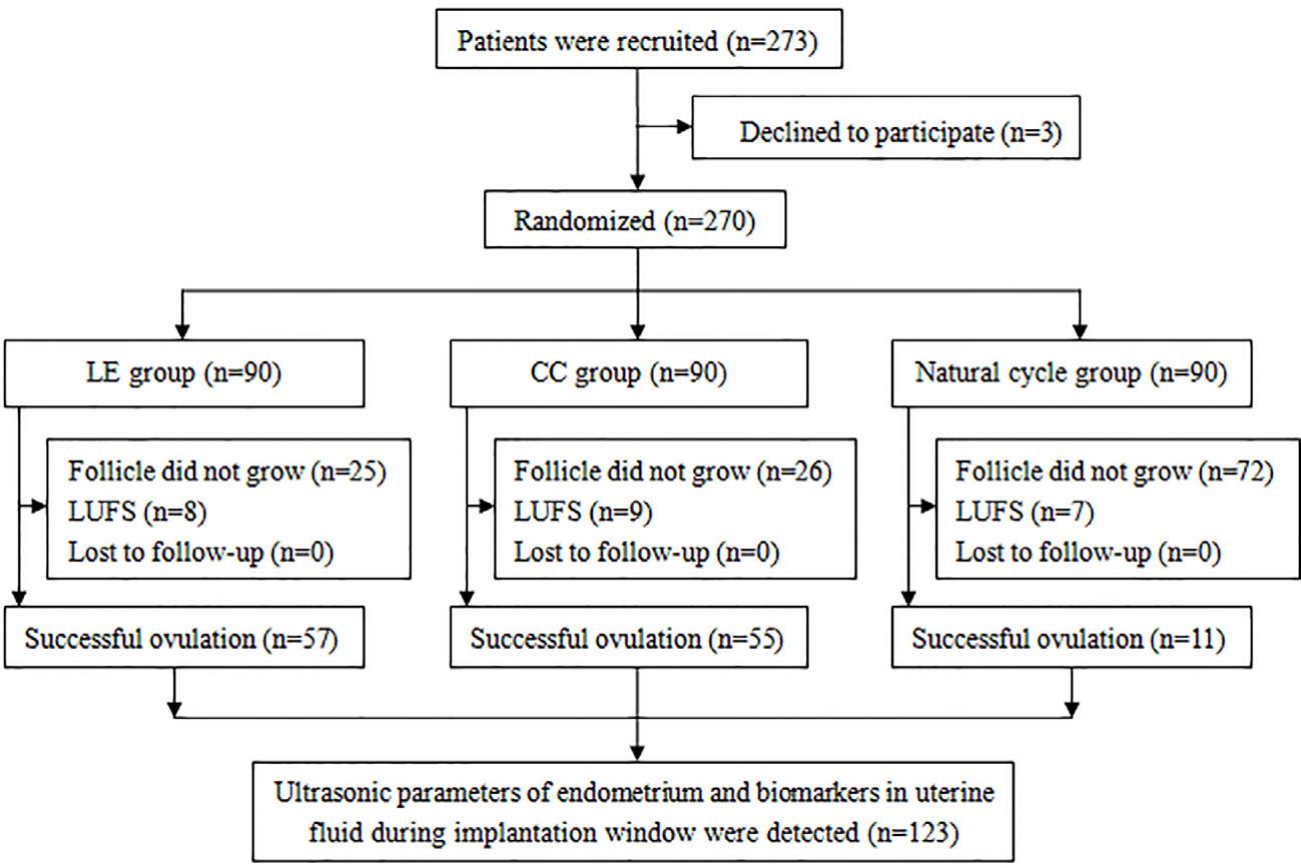
Flow chart of the study. (LUFS, luteinized unruptured follicle syndrome).

Two hundred and seventy-three PCOS patients were enrolled in this study, of which three patients were excluded. Hence, 270 patients were randomly assigned into three groups. There were 57, 55, and 11 patients with successful ovulation in the LE group, CC group, and natural cycle group, respectively. Endometrial ultrasonic parameters, integrin αvβ3, and VEGF in uterine fluid during the implantation window were tested (**Figure 1**).

### Basic Characteristic of Participants

**Table 1** indicates the basic data of patients among the three groups. The data show that no statistically significant differences were observed in the basic data of study subjects among the three groups (*P*>0.05).

**Table 1:** Basic data of study subjects among the three groups.

Characteristics	LE group (n=90)	CC group (n=90)	Natural cycle group (n=90)	*P* value^a^
Age (years)^b^	28.5 ± 7.6	28.3 ± 7.5	29.1 ± 6.1	0.315
Weight (kg)^b^	63.2 ± 8.7	61.8 ± 9.6	61.5 ± 10.4	0.242
BMI (kg/m^2^)^b^	24.9 ± 8.4	25.3 ± 7.9	24.7 ± 6.6	0.346
Waist circumference (cm)^b^	83.9 ± 10.9	81.6 ± 11.3	82.3 ± 11.4	0.138
Hip circumference (cm)^b^	95.1 ± 10.1	94.3 ± 9.9	96.4 ± 10.2	0.615
Waist-to-hip ratio^b^	0.9 ± 0.4	0.9 ± 0.2	0.8 ± 0.3	0.527
Basal concentration^b^				
FSH (mIU/mL)	7.3 ± 1.6	6.8 ± 1.2	7.2 ± 1.9	0.679
LH (mIU/mL)	10.2 ± 2.1	10.9 ± 1.9	11.3 ± 2.3	0.051
PRL (ng/mL)	14.2 ± 4.0	14.9 ± 4.9	13.6 ± 5.8	0.056
P (nmol/L)	1.6 ± 0.8	2.0 ± 0.7	1.9 ± 0.9	0.598
E_2_ (pmol/L)	59.1 ± 10.2	65.3 ± 12.8	61.9 ± 12.5	0.779
T (nmol/L)	1.5 ± 0.7	1.4 ± 0.6	1.3 ± 0.8	0.094
AMH (ng/mL)^b^	2.5 ± 0.6	2.7 ± 0.8	2.8 ± 0.9	0.213
Infertility duration (years)^b^	2.4 ± 0.7	2.3 ± 0.6	2.0 ± 0.5	0.125
Family history^c^				
Diabetes mellitus [n (%)]	7 (7.8)	4 (4.4)	6 (6.7)	0.644
Hypertension [n (%)]	9 (10.0)	8 (8.9)	10 (11.1)	0.884

^a^Variance analysis, chi-square test or Fisher’s exact test among the three groups. ^b^Data given as mean ± SD. ^c^Data given as number (%). FSH, follicle stimulating hormone; LH, luteinizing hormone; PRL, prolactin; P, progesterone; E_2_, estradiol; T, testosterone.

### Follicular Development and Ovulation Rate

**Table 2** reveals that the number of dominant follicles, number of ovulations, and successful ovulation rate in the LE group and CC group were significantly higher compared with the natural cycle group (*P*<0.05), but the above indicators did not differ between the LE group and CC group (*P*>0.05). Moreover, no statistically significant differences were observed in diameter of dominant follicle, dosage of hCG, and LUFS rate among the three groups (*P*>0.05).

**Table 2:** Comparison of follicular development and ovulation rate among the three groups.

Parameters	LE group (n=90)	CC group (n=90)	Natural cycle group (n=90)	*P* value^a^
Number of dominant follicle^b^	1.4 ± 0.5^★^	1.5 ± 0.6^★^	0.2 ± 0.1	0.022
Diameter of dominant follicle (mm)^b^	20.3 ± 6.1	20.4 ± 5.9	19.6 ± 5.8	0.897
Number of ovulation^b^	1.2 ± 0.5^★^	1.1 ± 0.4^★^	0.2 ± 0.1	0.025
Dosage of hCG (IU)^b^	8164.3 ± 128.5	8456.7 ± 139.1	9539.4 ± 124.6	0.052
Successful ovulation [n (%)]^c^	57 (63.3)^★^	55 (61.1)^★^	11 (12.2)	0.000
LUFS [n (%)]^c^	8 (8.9)	9 (10.0)	7 (7.8)	0.872

^a^Variance analysis or chi-square test among the three groups. ^b^Data given as mean ± SD. ^c^Data given as number (%). Vs. natural cycle group, ^★^P<0.05.

### Endometrial Receptivity

Among patients with successful ovulation in the three groups (57 patients in the LE group, 55 patients in the CC group, and 11 patients in the natural cycle group), the parameters of endometrial receptivity during embryo implantation were tested. The data in **Table 3** display that ET, EV, VI, FI, and VFI were significantly higher in the LE group, and integrin αvβ3 and VEGF concentrations in uterine fluid were also significantly higher in the LE group than in the CC group and natural cycle group (*P*<0.05), but no statistically significant differences were observed in the above parameters between the CC group and natural cycle group (*P*>0.05). In addition, the data show that no statistically significant differences were observed in uterine PI and RI among the three groups (*P*>0.05).

**Table 3:** Comparison of endometrial receptivity among the three groups.

Parameters	LE group (n=57)	CC group (n=55)	Natural cycle group (n=11)	*P* value^a^
Ultrasonic parameters				
Uterine PI	2.0 ± 0.6	2.2 ± 0.7	2.1 ± 0.6	0.676
Uterine RI	0.8 ± 0.2	0.8 ± 0.3	0.8 ± 0.2	0.890
ET (mm)	9.7 ± 2.5^★▲^	6.8 ± 1.9	6.2 ± 1.9	0.035
EV (cm^3^)	3.8 ± 0.9^★▲^	2.6 ± 0.7	2.7 ± 0.8	0.042
VI (%)	2.3 ± 0.8^★▲^	1.2 ± 0.5	1.3 ± 0.6	0.039
FI (0-100)	25.7 ± 6.0^★▲^	17.4 ± 5.7	15.9 ± 4.7	0.028
VFI (0-100)	0.6 ± 0.2^★▲^	0.3 ± 0.1	0.2 ± 0.1	0.031
Biomarkers (pg/mL)				
Integrin αvβ3	29.2 ± 10.3^★▲^	13.5 ± 7.1	8.5 ± 2.6	0.019
VEGF	35.6 ± 11.2^★▲^	17.9 ± 9.4	9.2 ± 3.8	0.013

Data given as mean ± SD. ^a^Variance analysis among the three groups. Vs. natural cycle group, ^★^P<0.05. Vs. CC group, ^▲^P<0.05.

### Pregnancy Rates

**Figure 2:**
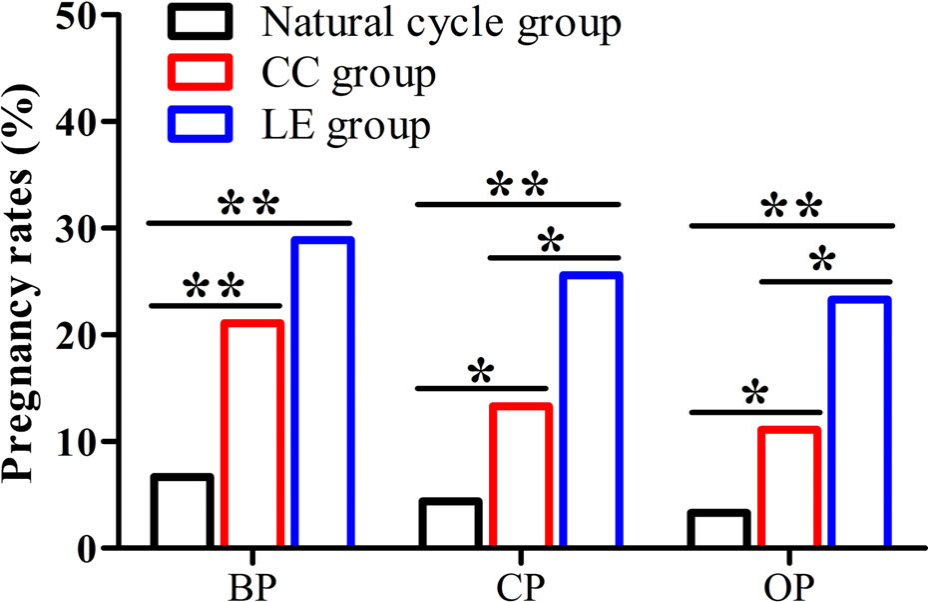
Pregnancy rates among the three groups. (BP, biochemical pregnancy; CP, clinical pregnancy; OP, ongoing pregnancy), *P < 0.05, **P < 0.01.

The data from **Figure 2** present that biochemical pregnancy rate, clinical pregnancy rate, and ongoing pregnancy rate of the LE group and CC group were significantly higher compared with the natural cycle group (28.9% or 21.1% vs. 6.7%, 25.6% or 13.3% vs.4.4%, 23.3% or 11.1% vs.3.3%) (*P*<0.05). In addition, clinical pregnancy and ongoing pregnancy rates of the LE group were significantly higher than that of the CC group (25.6% vs. 13.3%, 23.3% vs. 11.1%) (*P*<0.05).

### Relationship Between Endometrial Receptivity Parameters and Pregnancy

The patients with successful ovulation in this study were divided into four groups according to whether they were pregnant; 72 patients were in the no pregnancy group, 51 patients were in the biochemical pregnancy group, 39 patients were in the clinical pregnancy group, and 34 patients were in the ongoing pregnancy group. The data from **Table 4** suggest that endometrial VI, FI and VFI, integrin αvβ3, and VEGF concentrations in the uterine fluid of all pregnancy groups were significantly higher compared with the no pregnancy group (*P*<0.05). Moreover, the above parameters in ongoing pregnancy were significantly higher than that of the biochemical pregnancy group (*P*<0.05). However, no statistically significant differences were observed in uterine PI and RI, ET, and EV among the four groups (*P*>0.05).

**Table 4:** Comparison of endometrial receptivity parameters among the four groups.

Parameters	No pregnancy group (n=72)	Biochemical pregnancy group (n=51)	Clinical pregnancy group (n=39)	Ongoing pregnancy group (n=34)	*P* value^a^
Ultrasonic parameters					
Uterine PI	2.2 ± 0.5	2.1 ± 0.5	2.0 ± 0.6	2.1 ± 0.7	0.587
Uterine RI	0.8 ± 0.3	0.8 ± 0.3	0.8 ± 0.3	0.8 ± 0.2	0.324
ET (mm)	9.6 ± 2.3	9.9 ± 2.9	10.2 ± 3.0	9.9 ± 2.8	0.225
EV (cm^3^)	2.8 ± 0.8	2.9 ± 0.9	2.9 ± 0.8	3.0 ± 0.9	0.210
VI (%)	1.5 ± 0.6	2.2 ± 0.8^★^	2.5 ± 0.8^★^	2.9 ± 0.9^★▲^	0.044
FI (0-100)	14.4 ± 4.7	20.5 ± 5.1^★^	23.8 ± 5.7^★^	28.6 ± 5.9^★▲^	0.036
VFI (0-100)	0.2 ± 0.1	0.5 ± 0.2^★^	0.6 ± 0.3^★^	0.8 ± 0.4^★▲^	0.017
Biomarkers (pg/mL)					
Integrin αvβ3	11.2 ± 3.9	26.8 ± 7.3^★^	32.6 ± 7.4^★^	40.3 ± 10.2^★▲^	0.015
VEGF	16.7 ± 5.1	29.5 ± 9.4^★^	41.5 ± 10.9^★^	50.2 ± 11.5^★▲^	0.020

Data given as mean ± SD. ^a^Variance analysis among the three groups. Vs. no pregnancy group, ^★^P<0.05. Vs. biochemical pregnancy group, ^▲^P<0.05.

### Predictive Value for Pregnancy During Implantation Window

**Figure 3:**
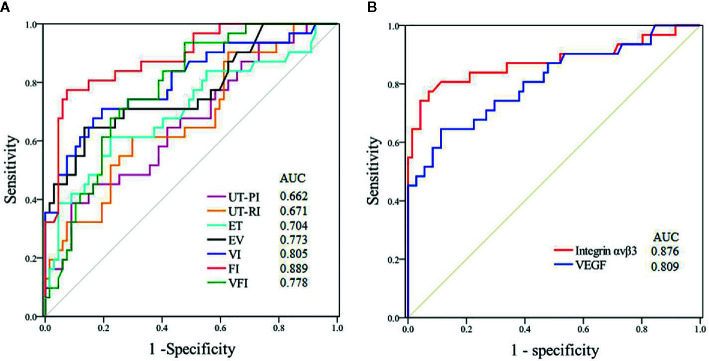
ROC curves of the predictive value for pregnancy during the implantation window in patients with PCOS. (UT-PI, PI of uterine artery; UT-RI, RI of uterine artery RI). **(A)** Ultrasonic parameters. **(B)** Biomarkers.

**Figure 3** demonstrates the predictive value of various parameters during the implantation window for pregnancy in PCOS patients. The best ultrasonic parameter for predicting pregnancy was endometrial FI (AUC=0.889); the cut-off of 22.9 provided a sensitivity of 92.7% and a specificity of 62.6%. The data show that integrin αvβ3 in uterine fluid during the implantation window had better predictive value (AUC=0.876) compared with VEGF; the cut-off of 29.6 pg/mL provided a sensitivity of 92.4% and a specificity of 54.8%.

## Discussion

Ovulation induction is regarded as an important therapeutic method for PCOS women with infertility. Previous studies reported that the ovulation rate of CC in PCOS women was 70%–80%. Nevertheless, the pregnancy rate was relatively low (25%–60%) (2). In addition to the side effects on cervical mucus (15), the main reason is that CC impaired endometrial development, which resulted in endometrial thinning and lower receptivity in PCOS women (16). Furthermore, approximately 15%–40% of women with PCOS are resistant to CC for ovulation induction (17). Because LE does not affect the central feedback mechanisms, they remain intact, making it superior to CC in ovulation induction. Hence, LE is recommended as first-line medication for ovulation induction in PCOS women according to the evidence-based medical evidence guidelines released internationally in 2018 (18).

Many studies were conducted to compare ovulation rates between CC and LE in women with PCOS. Literature has reported that LE was associated with higher cumulative ovulation rates among infertile patients with PCOS compared with CC (19). A recent meta-analysis of RCTs showed that LE significantly increased ovulation rates in women with PCOS (20). However, data in our study showed that the number of dominant follicles, number of ovulations, and successful ovulation rates in the LE group and CC group were significantly higher compared with the natural cycle group, but the above indicators did not differ between the LE group and CC group. The diverse outcomes perhaps related to the study design, different cycles, and dosages of LE and CC, race, and pre-treatment.

The endometrium has only a very short period of maximum sensitivity for embryo implantation in the normal menstrual cycle, about 7 to 9 days after ovulation. This period is defined as the window of implantation, and is the most appropriate time for embryo implantation (21). Infertility in PCOS women is related to anovulation and endometrial dysfunction. Both appropriate ET and adequate endometrial blood perfusion are crucial to embryo implantation. Therefore, the angiogenesis is active and the endometrial blood flow is increased in the early stage of embryo implantation, which offers support for pregnancy. With advances in diagnostic ultrasonography, several ultrasonographic indicators have been used to estimate endometrial receptivity, including uterine arterial blood flow, endometrial blood flow, and vascularisation parameters (22).

At present, 3-D energy Doppler ultrasonic scanning has been applied to estimate endometrial receptivity (23 **)**. Scholars proved that this technology has better predictive value for endometrial receptivity than 2-D ultrasound, which has been used for *in vitro* fertilization-embryo transfer (IVF-ET) (24). Yaman et al. reported that 3-D energy Doppler offers a unique tool to reflect the vascular structure of the whole endometrium and the supply of blood flow, which could be used as a predictor of pregnancy in patients who accepted assisted reproductive technology (25). Our findings indicate that patients in the LE group had significantly higher ET, EV, VI, FI, and VFI than patients in the CC group. Selim et al. found that ET was significantly thicker in the LE cycles compared to CC cycles (26), which were similar to our results. Roy et al. pointed out that the effect of LE displayed a better endometrial response and pregnancy rate than CC (27). Similarly, Al-Obaidi et al. reported that the application of LE in PCOS patients had a better curative effect on endometrial receptivity compared with CC (28). Additionally, the data in our study revealed that the endometrial VI, FI, and VFI of all pregnancy groups were significantly higher than that of the no pregnancy group, and the above indicators in ongoing pregnancy were significantly higher than those of the biochemical pregnancy group. In conclusion, the data in our study revealed that the endometrial receptivity during the implantation window in LE is superior to CC for ovulation induction among PCOS women.

Uterine fluid includes a variety of cytokines, which provide a suitable microenvironment for embryo implantation (29). Previous studies confirmed that intrauterine fluid aspiration during the window of implantation did not affect the success rate of pregnancy in women seeking pregnancy, such as patients who accepted intrauterine insemination or IVF-ET (30, 31). In addition, intrauterine fluid aspiration also had no effect on pregnancy in infertile patients with endometriosis, or patients with idiopathic infertility or luteal phase deficiency (32). Similar to previous studies, the safety of this method has been proven in our previous studies, which selected fertile women and patients with unexplained infertility as subjects (33). Data in our study revealed that integrin αvβ3 and VEGF concentrations in uterine fluid were significantly higher in the LE group compared with the CC group and natural cycle group. Integrin αvβ3 and VEGF concentrations in the uterine fluid of all pregnancy groups were significantly higher compared with the no pregnancy group. Additionally, integrin αvβ3 and VEGF concentrations in ongoing pregnancy were significantly higher compared with the biochemical pregnancy group. The concentrations of biochemical indicators in endometrial tissue and uterine fluid are related to embryo implantation. Boomsma et al. revealed that cytokines secreted by the endometrium provide a non-invasive method for evaluating endometrial receptivity and predicting embryo implantation (34).

According to the results of this study, clinical pregnancy and ongoing pregnancy rates of the LE group were significantly higher than in the CC group, which was consistent with previous studies. Wang et al. pointed out that LE improves clinical pregnancy and live birth rate compared to CC in women with PCOS (35). In another study, Roque et al. compared the results of CC versus LE for ovulation induction in PCOS women, and reported that LE was superior in pregnancy rates and live birth rates (36). Similarly, a recent study by Hu et al. found that LE significantly increased pregnancy rates and live birth rates in women with PCOS (20). Differences in pregnancy rates between patients with PCOS taking LE or CC may be explained by different endometrial receptivity during the implantation window, in addition to the changes of cervical mucus. Furthermore, our research also assessed the predictive value for pregnancy of observed indicators; data displayed that endometrial FI during the implantation window had the highest predictive value for pregnancy in patients with PCOS, the cut-off of 22.9 provided a sensitivity of 92.7% and a specificity of 62.6%. Integrin αvβ3 in uterine fluid during the implantation window had better predictive value than VEGF; the cut-off of 29.6 pg/mL provided a sensitivity of 92.4% and a specificity of 54.8%.

The present study still has some limitations. First, the RCT study was done in a single center. Additionally, the evaluative parameters were limited to one ovulation cycle, and live birth rate was not followed up. Second, endometrial receptivity is associated with many factors, including insulin resistance, hyperinsulinemia, thyroid hormone levels, abnormal immune-related indicators, haematologic parameters, and other cytokines. Therefore, multi-center studies with large sample sizes should be designed to verify the results of this research in the future.

## Conclusion

Endometrial receptivity during the implantation window of LE for ovulation induction is superior to CC in PCOS women, which may be associated with higher clinical pregnancy and ongoing pregnancy rates. Endometrial FI and integrin αvβ3 in uterine secretion during the implantation window could be preferable non-invasive predictor markers for pregnancy.

## Data Availability Statement

All datasets generated for this study are included in the article/ supplementary material.

## Ethics Statement

The studies involving human participants were reviewed and approved by First Affiliated Hospital of Xi’an Jiaotong University Institutional Review Board. The patients/participants provided their written informed consent to participate in this study. Written informed consent was obtained from the individual(s) for the publication of any potentially identifiable images or data included in this article.

## Author Contributions

LW and XY contributed to the conception and design of the study. SL provided participants. FL and EB participated in data collection and patients’ follow-up. All authors contributed to the article and approved the submitted version.

## Funding

This study was supported by the Natural Science Basic Research Program of Shaanxi (Program No.2019JM-569) and by the Institutional Foundation of the First Affiliated Hospital of Xi’an Jiaotong University (Program No.2019ZYTS-03).

## Conflict of Interest

The authors declare that the research was conducted in the absence of any commercial or financial relationships that could be construed as a potential conflict of interest.
